# Effect of Changing Gloves Before Placental Extraction on Incidence of Postcesarean Endometritis

**DOI:** 10.1155/S106474499600004X

**Published:** 1996

**Authors:** Mark A. Turrentine, Tracey A. Banks

**Affiliations:** 1 Department of Obstetrics, Gynecology, and Reproductive Sciences University of Texas Health Science Center Houston TX USA; 2 Department of Obstetrics and Gynecology MacGregor Medical Association 6410 Fannin, Suite 200-A Houston TX 77030 USA

## Abstract

*Objective:* We sought to determine if changing the surgeon's gloves after delivery of the infant and prior to manual placental removal decreases the incidence of postcesarean endometritis.

*Methods:* Laboring women undergoing cesarean delivery between September 1, 1994, and August 31, 1995, were prospectively randomized into either a change or no-change glove group. In the change-glove group, the surgeon's gloves were changed after delivery of the infant and before manual removal of the placenta. All patients enrolled received a single prophylactic dose of an IV antibiotic after clamping of the umbilical cord. Endometritis was diagnosed by an oral temperature of ≥38℃ on 2 occasions at least 6 h apart and >24 h after delivery, uterine tenderness, peripheral blood leukocytosis (≥15,000 cells/ml), and the exclusion of other foci of infection. In order to detect a reduction in endometritis from 14% to 2%, at *P* < 0.05 with 80% power, we needed 95 patients in each group.

*Results:* Two hundred twenty-eight women were randomized to 2 groups: 113 were in the change group and 115 in the no-change group. No significant differences were noted between the groups with respect to demographics, duration of labor, length of ruptured membranes, number of vaginal examinations, duration of internal monitoring, length of surgery, blood loss, or infant weight. There was no decrease in the incidence of endometritis between the change group (17.7%) and the no-change group (15.7%) (relative risk 1.1, 95% confidence interval 0.75–1.47).

*Conclusions:* In this study, the incidence of postcesarean endometritis was not decreased by changing the surgeon's gloves after delivery of the infant but before placental extraction.


Infectious Diseases in Obstetrics and Gynecology 4:16–19 (1996)
© 1996 Wiley-Liss, Inc.

### Effect of Changing Gloves Before Placental Extraction on Incidence of Postcesarean Endometritis

**Mark A. Turrentine and Tracey A. Banks**

*Department of Obstetrics, Gynecology, and Reproductive Sciences, University of Texas Health Science Center, Houston, TX*

### Abstract

***Objective:*** **We sought to determine if changing the surgeon's gloves after delivery of the infant and prior to manual placental removal decreases the incidence of postcesarean endometritis.**

***Methods:*** **Laboring women undergoing cesarean delivery between September 1, 1994, and August 31, 1995, were prospectively randomized into either a change or no-change glove group. In the change-glove group, the surgeon's gloves were changed after delivery of the infant and before manual removal of the placenta. All patients enrolled received a single prophylactic dose of an IV antibiotic after clamping of the umbilical cord. Endometritis was diagnosed by an oral temperature of ≥38°C on 2 occasions at least 6 h apart and >24 h after delivery, uterine tenderness, peripheral blood leukocytosis (≥15,000 cells/ml), and the exclusion of other foci of infection. In order to detect a reduction in endometritis from 14% to 2%, at $P < 0.05$ with 80% power, we needed 95 patients in each group.**

***Results:*** **Two hundred twenty-eight women were randomized to 2 groups: 113 were in the change group and 115 in the no-change group. No significant differences were noted between the groups with respect to demographics, duration of labor, length of ruptured membranes, number of vaginal examinations, duration of internal monitoring, length of surgery, blood loss, or infant weight. There was no decrease in the incidence of endometritis between the change group (17.7%) and the no-change group (15.7%) (relative risk 1.1, 95% confidence interval 0.75–1.47).**

***Conclusions:*** **In this study, the incidence of postcesarean endometritis was not decreased by changing the surgeon's gloves after delivery of the infant but before placental extraction.**
© 1996 Wiley-Liss, Inc.

Key words

Puerperal infection, cesarean delivery, bacterial contamination

Cesarean delivery is the greatest attributable risk for puerperal endometritis.[1] The pathophysiology of postcesarean endometritis is dependent upon a complex balance between host defense mechanisms and bacterial virulence factors.[2] With the advent of antibiotic prophylaxis at the time of surgery, significant decreases in the incidence of postcesarean endometritis have been achieved.[3] Yet, despite the use of antibiotic prophylaxis, postcesarean endometritis may develop in up to 31% of patients undergoing cesarean delivery.[3] Recent observations suggest an association between manual removal of the placenta at the time of cesarean delivery and a 7-fold increase in the incidence of postcesarean endometritis.[4] It has been suggested that the observed increase in postcesarean endometritis may result from the contamination of the endometrial cavity by bacteria on the surgeon's gloves during

Address correspondence/reprint requests to Dr. Mark A. Turrentine, Department of Obstetrics and Gynecology, MacGregor Medical Association, 6410 Fannin, Suite 200-A, Houston, TX 77030.

**Presented at the 44th Annual Clinical Meeting of the American College of Obstetricians and Gynecologists, April 29–May 1, 1996, Denver.**

Received November 27, 1995
Accepted March 27, 1996

Clinical Study the process of extracting the placenta.[5] In support of this explanation is that, in laboring women undergoing cesarean delivery, bacterial contamination of the surgeon's gloves following infant delivery has been shown.[5] Therefore, for minimizing bacterial contamination of the uterine cavity, it has been proposed that the gloves on the surgeon's hands be replaced immediately following delivery of the infant.[5] The present investigation was undertaken to determine if changing the surgeon's gloves after delivery of the infant and prior to placental extraction would decrease the incidence of postcesarean endometritis.

### SUBJECTS AND METHODS

This study was approved by the institutional review board of the University of Texas Health Science Center—Houston. Women in labor undergoing cesarean delivery were eligible with the following exclusion criteria: 1) intra-amniotic infection or 2) prophylactic intrapartum or postpartum antibiotics. A randomized prospective trial was conducted from September 1, 1994, through August 31, 1995, enrolling patients undergoing cesarean delivery at Hermann Hospital, Houston, TX.

Labor was defined as painful regular contractions leading to cervical change. The onset of labor was defined as the time of admission to the labor floor. Randomization was accomplished by a computer-generated random-number table. Each eligible patient was randomly assigned to either a change-glove group or a no-change group. In the change-glove group, the surgeon's gloves were changed after delivery of the infant but before manual extraction of the placenta.

Standard abdominal preparation and aseptic surgical techniques were utilized at surgery. An attending physician or chief resident was scrubbed on all surgeries. All patients received a single prophylactic dose of an IV antibiotic after clamping of the umbilical cord. The placenta was manually removed following delivery of the infant. The uterus was exteriorized and the incision closed. The patients received routine postoperative care and evaluations for the outcome variable of endometritis.

Endometritis was diagnosed by a fever of ≥38°C on 2 occasions at least 6 h apart and >24 h after delivery, uterine tenderness, peripheral blood leukocytosis (≥15,000 cells/ml), and the absence of other foci of infection. Upon completion of the study, the medical records were computer searched to determine if any study patients not diagnosed with endometritis and discharged home were readmitted within 2 weeks of discharge with the diagnosis of endometritis.

Dichotomous variables were compared using $\chi^2$ analysis. Continuous variables were compared using either the t-test, Mann-Whitney rank sum, or median test, as appropriate. Power calculations were based on a postcesarean endometritis rate of 14% in our population.[6] When compared with manual removal of the placenta, spontaneous placental extraction results in a 7-fold reduction in the incidence of postcesarean endometritis.[4] To detect a similar 7-fold reduction, i.e., 14% to 2%, in the incidence of postcesarean endometritis by changing the surgeon's gloves at $P < 0.05$ with 80% power, we needed 95 patients in each group.

### RESULTS

From September 1, 1994, through August 31, 1995, 4,741 deliveries occurred, with 913 women undergoing cesarean delivery. Of the cesarean deliveries, 292 (32.0%) were not in labor, 101 (11.1%) were diagnosed with intra-amniotic infection, 197 (21.6%) received either prophylactic intrapartum or postpartum antibiotics, and 95 (10.4%) failed to be entered into the study. Remaining were 228 eligible patients, with 113 randomized to the change group and 115 to the no-change group.

The demographic characteristics of each randomized study group is shown in Table 1. There were no significant differences between the groups in age, racial distribution, incidence of nulliparity, type of medical insurance, or estimated gestational age.

The intrapartum and delivery variables between the change and no-change groups are shown in Table 2. There were no significant differences in indications for cesarean, duration of labor, duration of ruptured membranes or incidence of ruptured membranes >6 h, incidence or duration of internal monitoring, number of vaginal examinations, type of uterine incision, type of prophylactic antibiotic, operating time, estimated blood loss, infant birth weight, or the percent of infants weighing <2,500 g.

No decrease was seen in the incidence of postcesarean endometritis between the change glove

**TABLE 1. Demographic characteristics at enrollment**

| | Change glove (N = 113) | No change (N = 115) |
|---|---|---|
| Age (years)[a] | 25.6 ± 6.6 | 26.4 ± 6.6 |
| Race (%) | | |
| Black | 53.1 | 44.3 |
| Hispanic | 22.1 | 23.5 |
| White | 21.2 | 22.6 |
| Other | 3.5 | 9.6 |
| Type of insurance (%) | | |
| Government | 51.5 | 49.6 |
| Health maintenance organization | 28.3 | 36.5 |
| Commercial | 13.2 | 11.3 |
| Nonresource | 7.1 | 2.6 |
| Incidence of nulliparity (%) | 61.9 | 50.4 |
| Gestational age (weeks)[a] | 38.2 ± 3.1 | 38.3 ± 3.4 |

[a]Results are mean ± standard deviation. No differences were statistically significant.

**TABLE 2. Intrapartum and delivery variables[a]**

| | Change glove (N = 113) | No change (N = 115) |
|---|---|---|
| Indication for cesarean (%) | | |
| Arrest disorder | 38.9 | 42.6 |
| Malpresentation | 29.2 | 19.1 |
| Fetal distress | 25.7 | 30.4 |
| Others | 6.2 | 7.8 |
| Duration of labor (h) | 8.9 ± 6.6 | 9.1 ± 6.8 |
| Rupture of membranes | | |
| Duration (h) | 8.2 ± 9.4 | 7.6 ± 8.7 |
| >6 h (%) | 42.5 | 52.2 |
| Internal monitoring | | |
| Incidence (%) | 60.2 | 58.3 |
| Duration (h) | 6.1 ± 4.3 | 5.4 ± 3.7 |
| Number of vaginal examinations[b] | 5 | 6 |
| Uterine incision | | |
| Low segment transverse (%) | 91.1 | 95.7 |
| Type of antibiotic prophylaxis (%) | | |
| Cefotetan | 92.9 | 93.0 |
| Other | 7.1 | 7.0 |
| Operating time (min) | 45.5 ± 15.7 | 45.3 ± 16.6 |
| Decrease in hemoglobin (g/dl) | 1.9 ± 0.9 | 1.7 ± 1.1 |
| Infant birth weight (g) | 3,006 ± 868 | 3,110 ± 825 |
| Infants <2,500 g (%) | 25.7 | 18.3 |

[a]All values are mean ± standard deviation unless otherwise indicated. No differences were statistically significant.
[b]Median.

group (17.7%) vs. the no-change group (15.7%) (relative risk 1.1, 95% confidence interval 0.75–1.47).

The outcomes of the 95 laboring women who were not enrolled in the study were analyzed. In none of these patients were the surgeon's gloves changed after delivery of the infant. The rate of endometritis was 14.7%, which was not statistically different ($P = 0.74$) from the overall rate of endometritis (16.7%) in the 228 women randomized to the study.

### DISCUSSION

Cesarean delivery predisposes women to intrauterine infection.[1] During labor and abdominal delivery, the endometrial cavity is contaminated with large numbers of bacteria.[7] Prophylaxis with antimicrobial therapy reduces the bacterial inoculum below a critical level and permits the host's immune system to exert a protective effect.[8] Antibiotic prophylaxis has significantly reduced the incidence of postcesarean endometritis.[3] However, postcesarean endometritis occurs in an average of 12% of women despite prophylaxis with antibiotics.[3]

McCurdy et al.[4] observed that the manual removal of the placenta at cesarean delivery is associated with a significant increase in postcesarean endometritis compared with spontaneous removal. It is postulated that manual extraction of the placenta at the time of cesarean delivery results in either tissue trauma, maternal autotransfusion of bacterially contaminated blood before implantation-site involution, surgical contamination, or excessive blood loss which yields a higher rate of endometritis.[4]

Other investigators have proposed that the findings of McCurdy et al.[4] may be that manual extraction of the placenta either impairs local host defenses or allows the introduction of bacteria directly into the endometrial cavity which increases the likelihood of postoperative infection.[5] In favor of the latter hypothesis was the study of Yancey et al.[5] in which the gloves of surgeons were cultured for aerobic and anaerobic organisms immediately before and after delivery of the infants in 25 women having scheduled or unscheduled cesarean delivery. In the laboring patients, nonstaphylococcal bacteria were isolated in 79% of the cases compared with 9% in nonlaboring women. They concluded that, in laboring patients with ruptured membranes, delivery of the infant results in contamination of the surgeon's gloves with pathogenic bacteria. They recommended that the surgeon's gloves be replaced immediately following delivery of the neonate.

This precaution would minimize the introduction of bacteria to the uterus and possibly reduce the risk of postoperative infection. However, they acknowledged that this hypothesis needs the results of a randomized investigation.

In the present study, we found no significant decrease in the incidence of postcesarean endometritis when the surgeon changed gloves after delivery of the infant but prior to manual removal of the placenta. Several hypotheses may be evoked to explain our findings. First, the independent variables which affect the incidence of postcesarean endometritis may have differed between the control and study groups. Etiologic factors for postcesarean endometritis such as bacterial contamination of the endometrial cavity, impairment of host-defense mechanisms following disruption of the blood supply to the uterus, introduction of foreign material such as suture, and possible suppression of host-defense mechanisms because of poor nutrition during the immediate postoperative period were not examined.[2] However, various maternal demographics and characteristics of parturition that have been shown to impact the incidence of postcesarean endometritis[1–4,7,9] were not significantly different between the groups.

Second, the magnitude of the decrease in postcesarean endometritis that was anticipated to be measured by changing the surgeon's gloves was too large. McCurdy et al.[4] showed a 7-fold decrease (23% to 3%) in postcesarean endometritis when spontaneous removal of the placenta was compared with manual removal of the placenta at the time of cesarean delivery. The incidence of postcesarean endometritis of 3% seen in the study by McCurdy et al.[4] is similar to the 2% reported in nonlaboring patients without ruptured membranes undergoing cesarean delivery with antibiotic prophylaxis.[10] Reductions of a smaller magnitude may have been undetected by our study, but they are also less clinically significant.

Finally, bacterial inoculation of the endometrium from the contaminated surgeon's glove is not sufficient to either initiate a clinical infection or increase the amount of bacteria that may be present. Patients who fail antibiotic prophylaxis may have an incipient infection at the time of cesarean delivery which predisposes them to postcesarean endometritis.[11]

In conclusion, changing the surgeon's gloves after delivery of the infant and prior to manual removal of the placenta at cesarean delivery did not decrease the incidence of postcesarean endometritis.

### REFERENCES

1. Newton ER, Prihoda TJ, Gibbs RS: A clinical and microbiologic analysis of risk factors for puerperal endometritis. Obstet Gynecol 75:402–406, 1990.
2. Duff P: Pathophysiology and management of postcesarean endomyometritis. Obstet Gynecol 67:269–276, 1986.
3. Swartz WH, Grolle K: The use of prophylactic antibiotics in cesarean section. A review of the literature. J Reprod Med 26:595–609, 1981.
4. McCurdy CM, Magann EF, McCurdy CJ, Saltzman AK: The effect of placental management at cesarean delivery on operative blood loss. Am J Obstet Gynecol 167:1363–1367, 1992.
5. Yancey MK, Clark P, Duff P: The frequency of glove contamination during cesarean delivery. Obstet Gynecol 83:538–542, 1994.
6. Gonik B: Single- versus three-dose cefotaxime prophylaxis for cesarean section. Obstet Gynecol 65:189–193, 1985.
7. Gilstrap LC, Cunningham FG: The bacterial pathogenesis of infection following cesarean section. Obstet Gynecol 53:545–549, 1979.
8. Duff P: Prophylactic antibiotics for cesarean delivery: A simple cost-effective strategy for prevention of postoperative morbidity. Am J Obstet Gynecol 157:794–798, 1987.
9. Chang PL, Newton ER: Predictors of antibiotic prophylactic failure in post-cesarean endometritis. Obstet Gynecol 80:117–122, 1992.
10. Duff P, Smith PN, Keiser JF: Antibiotic prophylaxis in low-risk cesarean section. J Reprod Med 27:133–138, 1982.
11. Gonik B, Shannon RL, Shawar R, Costner M, Seibel M: Why patients fail antibiotic prophylaxis at cesarean delivery: Histologic evidence of incipient infection. Obstet Gynecol 79:179–184, 1992.
